# Effect of dairy products fortified with vitamin d_3_ on restless legs syndrome in women with premenstrual syndrome, abdominal obesity and vitamin d deficiency: a pilot study

**DOI:** 10.1186/s12905-024-03159-z

**Published:** 2024-07-30

**Authors:** Payam Sharifan, Toktam Sahranavard, Mohammad Rashidmayvan, Susan Darroudi, Mohammad Vahedi Fard, Kimia Mohammadhasani, Amin Mansoori, Saeid Eslami, Mohamad Safarian, Asma Afshari, Mohammad Bagherniya, Maryam Tayefi, Majid Khadem-Rezaiyan, Hamideh Ghazizadeh, Gordon Ferns, Mitra Rezaie, Majid Ghayour-Mobarhan

**Affiliations:** 1 Department of Nutrition, School of Medicine, Mashhad University of Medical Sciences, Mashhad, Iran; 2 Student Research Committee, School of Medicine, Mashhad University of Medical Sciences, Mashhad, Iran; 3 International UNESCO center for Health-Related Basic Sciences and Human Nutrition, Mashhad University of Medical Sciences, Mashhad, Iran; 4 Department of Nutrition, Food Sciences and Clinical Biochemistry, School of Medicine, Social Determinants of Health Research Center, Gonabad University of Medical Science, Gonabad, Iran; 5 Department of Applied Mathematics, School of Mathematical Sciences, Ferdowsi University of Mashhad, Mashhad, Iran; 6 Department of Biostatistics, School of Health, Mashhad University of Medical Sciences, Mashhad, Iran; 7 Department of Medical Informatics, School of Medicine, Mashhad University of Medical Sciences, Mashhad, Iran; 8 Department of Community Nutrition, School of Nutrition and Food Science, Food Security Research Center, Isfahan University of Medical Sciences, Isfahan, Iran; 9 Norwegian Center for e-health Research, University Hospital of North Norway, Tromsø, Norway; 10 Clinical Research Development Unit, Faculty of Medicine, Mashhad University of Medical Sciences, Mashhad, Iran; 11 Brighton and Sussex Medical School, Division of Medical Education, Brighton, UK; 12 Metabolic Syndrome Research Center, School of Medicine, Mashhad University of Medical Sciences, Mashhad, 99199-91766 Iran; 13 Vascular and Endovascular Surgery Research Center, Mashhad University of Medical Sciences, Mashhad, Iran

**Keywords:** Fortification, Restless legs syndrome, Premenstrual syndrome, Vitamin D, Low-fat dairy

## Abstract

**Backgrounds:**

Restless legs syndrome (RLS) is an unpleasant condition that affects the quality of life of patients. Its prevalence in increased in women with premenstrual syndrome (PMS). Vitamin D plays a key role in female reproduction through its impact on calcium homeostasis and neurotransmitters. We aimed to evaluate the effect of dairy products fortified with Vitamin D_3_ on RLS in women with PMS.

**Materials and methods:**

We conducted a 2.5-month, randomized, total-blinded clinical trial to evaluate the effectiveness of low-fat milk and yogurt fortified with vitamin D on RLS in women with PMS. Among 141 middle-aged women with abdominal obesity, 71 and 70 cases received fortified and non-fortified low-fat dairy products, respectively. All subjects completed a Symptoms Screening Tool (PSST) and RLS questionnaires.

**Results:**

The results showed that in the women with severe PMS (PSST > 28), serum levels of vitamin D increased significantly following vitamin D fortification. The mean restless legs score in the severe PMS subgroup (PSST > 28) was significantly lower after the intervention (*p* < 0.05. Serum Vitamin D levels significantly differed between intervention and control groups in all individuals (PSST < 19, PSST 19–28, and PSST > 28) (*p* < 0.05), but no significant differences were found between RLS scores of the intervention and control groups in the three PMS subgroups (*p* > 0.05).

**Conclusion:**

Fortifying dairy products with vitamin D_3_ can increase the serum levels of vitamin D and reduce the RLS severity in women with severe PMS, but not in other groups.

## Introduction

RLS is a widespread sensorimotor neurological disorder affecting many adults [1, 2]. This disease is characterized by an imperative urge to move the limbs, with an uncomfortable sensation in the ankles and calves at night, resulting in sleep disturbances [3, 4]. Furthermore, patients with RLS may experience lack of energy, depression, and irritability [5]. RLS is a significant clinical and public health issue, experienced by 5–10% of the European and North American population and also 1 to 4% of people from Asia [6]. RLS is mostly considered to be an idiopathic or hereditary condition (primary RLS), but it can also be secondary, caused by several other medical conditions [7]. RLS’s exact pathophysiology has not yet been fully elucidated, but may be related to dopamine system function. The successful therapeutic response to dopamine agonist use in RLS is the most robust evidence of this syndrome’s dopaminergic dysfunction hypothesis [8, 9]. Obesity may be a risk factor for RLS [10]. The association between obesity and lower expression of dopamine receptors may explain the association between obesity and RLS [11].

Sleep disturbances (e.g., RLS) are common for women in the late luteal phase and are related to depression, affective lability, anxiety, and other emotional symptoms known as PMS [12, 13]. PMS is a prevalent condition in modern society [14]. Mild to moderate PMS symptoms affect up to 75% of fertile women, and between 3 and 8% of women present severe symptoms, which may disturb their daily activities [15]. PMS begins with recurrent episodes of emotional, behavioral, and physical signs and symptoms that happen during the luteal stage of the menstrual cycle, and it ends with the onset of periodic bleeding [16]. Women with PMS may experience mood swings, irritability, fatigue, abdominal pain, constipation, nausea, changes in appetite, and weight gain [17]. Despite the improved understanding of the pathophysiology of PMS in recent years, its etiology remains unclear. The symptomatic cases mainly use oral contraceptive pills, serotonin reuptake inhibitors, and gonadotropin-releasing hormone agonists that may be capable bring relief. At the same time, their side effects have led to great concern [15, 18]. Furthermore, obesity may increase the risk of PMS by changing the function of neurotransmitters [19].

Doing exercise and consuming some vitamins and minerals, such as B6, B12, D, and magnesium can improve RLS [6, 20–22]. It is estimated that approximately 1 billion people have vitamin D insufficiency around the world [23]. The prevalence of vitamin D deficiency is high, particularly in women of the middle east, and moderate to severe in Iran [24]. Risk factors for vitamin D deficiency or insufficiency include limited direct sunlight exposure, aging, dark skin pigmentation, high body mass index (BMI), low dietary consumption of vitamin D, malabsorption conditions, some medicines (anticonvulsants, anti-tuberculous drugs), and hepatic or renal disorders [25]. Vitamin D has an important role in the function of the dopamine system. It has been shown that vitamin D rises the level of dopamine or the metabolites and supports dopaminergic nervous cells against toxic substances [26, 27]. Although many studies have examined the importance of vitamin D in dopamine function, its influence on RLS has not yet been determined [8]. Vitamin D receptors are found in ovarian tissue, endometrium, epithelial cells of the fallopian tube, placenta, and decidua, which explain why vitamin D is important in female reproduction [28, 29]. Data from several investigations indicate a possible involvement of vitamin D in reducing PMS risk. It seems it is primarily correlated with calcium concentration modulation, particular neurotransmitters, and sexual steroids [30, 31].

There is a lower possibility of developing PMS in women who receive high levels of dietary vitamin D and calcium [18]. Calcium is mainly present in milk. But vitamin D is also found fatty fish and fish oils, or it could be fortified in some foods like dairy products [32]. Hence, vitamin D supplementation/fortification has been supposed to control or even eradicate vitamin D deficiency [33].

Although the effects of vitamin D supplementation and calcium on PMS have previously been reviewed, there is a lack of information demonstrating the effectiveness of vitamin D in dairy products as a fortified ingredient. This research was undertaken to investigate the effectiveness of yogurt and low-fat milk fortified with 1500 IU Nano encapsulated vitamin D on RLS in women with PMS.

## Methods

### Study design

This was a pilot study is a sub-analysis of a survey of ultraviolet intake by nutritional approach (SUVNIA) study (trial registration: IRCT20101130005280N27, www.IRCT.ir, date of registration: 3.9.2018) which consisted of an intervention period of ten weeks to assess the efficacy of consumption of low-fat dairy products fortified by 1500IU nano encapsulated vitamin D_3_ on physical and mental aspects of health. The protocol study was published elsewhere [34]. This study was a parallel total-blind randomized clinical trial which was performed from January 2019 until March 2019.

### Participants

A power calculation determined that a sample size of 280 subjects (70 subjects in each group) was needed by considering 80% of power, an effect size of 0.5, and a potential dropout rate of 10% as a pilot study. Regarding the good contribution of participants, we increased the sample size up to 306 subjects.

Subjects were enrolled from Mashhad University of Medical Sciences (Mashhad-Iran) among students, staff, and their relatives who agreed to participate in the SUVINA study. The middle-aged adults (30 to 50 years old) with abdominal obesity were selected (*n* = 306). Among the participants who were included in the whole trial (*n* = 289), the number of women was 141 and subsequently entered into this pilot study. Body weight was measured to the nearest 0.1 kg with electronic scales. Height was measured using a wall stadiometer. A digital bio-impedance analyzer (Tanita BC 418, Japan) was used to analyze individuals body composition for fat-free mass, fat mass, and total body water. Body mass index (weight (kg)/height2 (m2)) was estimated. waist circumference was measured twice using a flexible nonelastic tape by a single experienced staff member. The measurement was taken at the midpoint between the iliac crest edge and the bottommost rib, at the end of a normal exhalation. Additionally, hip circumference was measured around the hips. According to the International Diabetes Federation (IDF) waist circumference (WC) ≥ 80 cm for women and ≥ 94 cm for men was considered Abdominal obesity [35]. All of the subjects completed a general information checklist, including demographic information, history of any diseases, and current treatments before starting the trial. A 2.5 months intervention was considered for the participants. In each intervention group, fortified milk or yogurt with vitamin D was received. The control group consumed non-fortified milk or yogurt. A three-day food records before, at the middle, and at the end of the study to control energy and vitamin D intake during the study. In this regard, we used a validated food frequency questionnaire [36]. Inclusion criteria were aged between 30 and 50, having approval for participation in the study. All individuals were interviewed in-person and one by one. Adherence to consumption has been evaluated through returning empty single used containers from participants. Also, we asked participants to consume products in front of the research staff as possible.

**Fig. 1:**
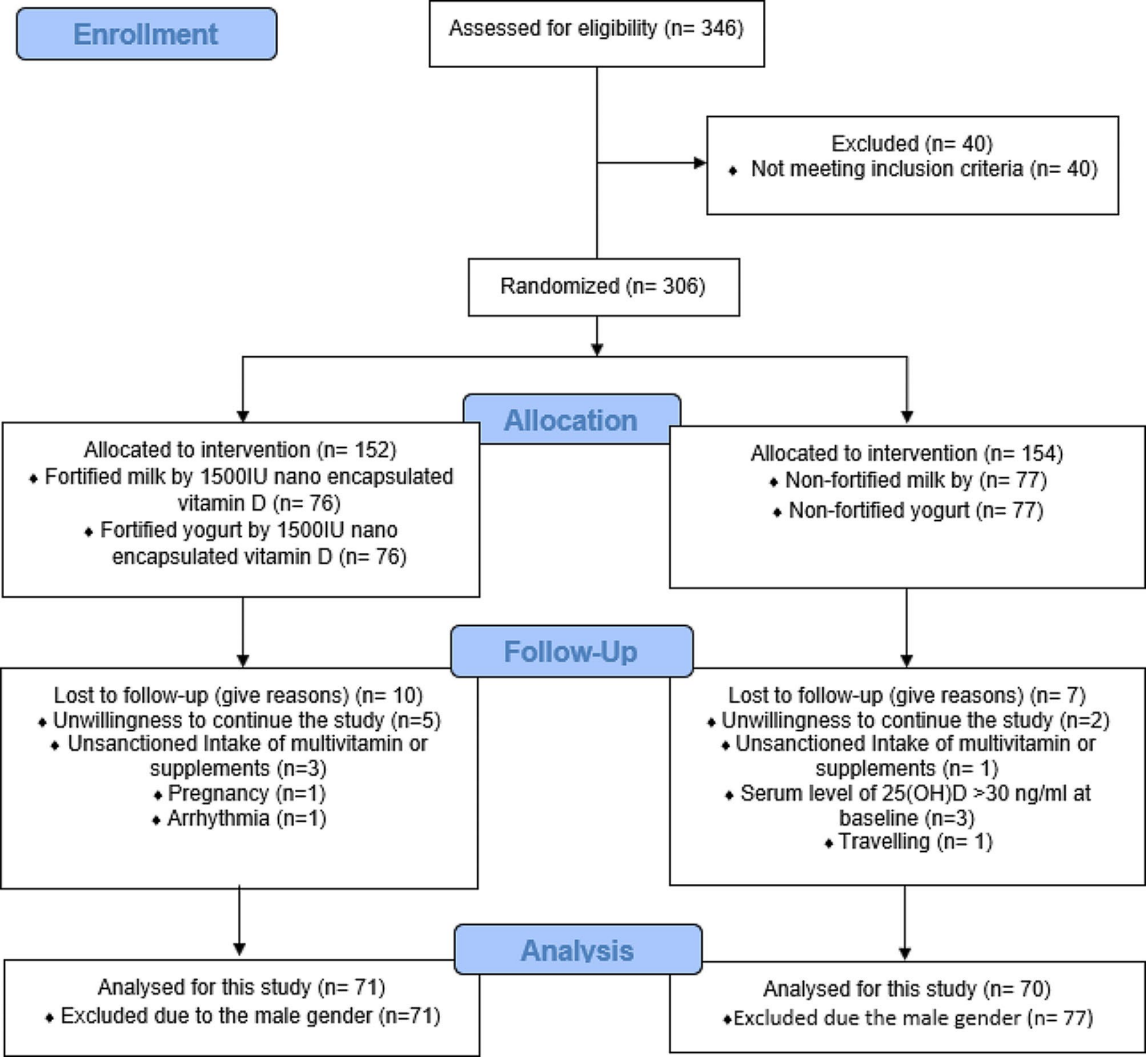
Flow chart of the study. ONSORT 2010 flow diagram

Other inclusion criteria were no planned change in weight during the study, no history of lactose intolerance or sensitivity, women who are not pregnant or lactating, not using supplements containing vitamin D or any medications with interaction with vitamin D (anticonvulsants, corticosteroids, sleeping medications, antidepressant, contraceptives) in 3 months before the trial (Fig. 1). All participants had an interview with a registered physician for their medical history.

Exclusion criteria were having sufficient levels of vitamin, taking any vitamin D supplements three months before the survey, consumption of multivitamins or supplements throughout the trial, having Chronic Liver Diseases, Cystic Fibrosis, or Crohn’s. All 141 women filled out the PSST questionnaire [37]. Also, the assessment of RLS was only conducted by a restless legs syndrome questionnaire [38].

### Procedure

The subjects were recruited in the month preceding the intervention (November 2018). We conducted a screening procedure consisting of three steps: (1) phone call, (2) in person visit at clinic, (3) visit for approval form. Then a skillful family physician collected a medical history and physical examination that included height, weight, and WC.

### Randomization and blinding

Stratified block assignment was done for desirable participants for the center and sex status with a ratio of 1:1:1:1 to receive fortified low-fat milk having 1500 IU Nano encapsulated vitamin D_3_ /serving (200mL/day), simple low-fat milk (200mL/day), fortified low-fat yogurt having 1500 IU Nano encapsulated vitamin D_3_ /serving (150 g/day) and simple low-fat yogurt (150 g/day) for ten weeks’ trial. An individual who did not participate in the collection of data, analysis, and reporting, carried out the random allocation.

For the placebo and intervention groups, closed envelopes including A or B labels were used. The envelopes were opened in order, in front of each participant. The assignment list remained secured by the faculty of medicine until the end of the study and did not provide any access to the researchers. Blinding was performed for subjects, statisticians, investigators, outcome evaluators, and staff who assigned participants to the groups (total blinding).

### Questionnaire assessment

The first endpoint was changed in restless legs symptoms using the validated RLS questionnaire [38] after the 2.5-month trial period. The restless legs syndrome questionnaire has ten items with five options; each item has zero to four points. The severity of the disorder is based on scores obtained in five categories: The severity of RLS is defined as follows: Very severe: 31 to 40 points, Severe: 21 to 30 points, Moderate: 11 to 20 points, Mild: 1 to 10 points, none: 0 points.

The secondary endpoint was PMS, assessed using the PSST validated questionnaire [37] after the ten weeks trial period. The PSST questionnaire consists of nineteen questions that examine the symptoms of PMS and its’ effect on the lives of individuals. The questionnaire consists of two parts. The first part consists of 14 questions on mood, physical, and behavioral symptoms, and the second part assesses the impact of these symptoms on the lives of the people. The severity of PSST is described as follows: Mild: <19 points, Moderate: 19 to 28 points, and Severe > 28 points.

### Laboratory measurements

Twenty milliliters of venous blood were taken from each participant pre and post intervention. Samples were assembled and centrifuged (3500 RPM, and 15 min), and then kept at -80◦C by an experienced technician. 25 (OH) vitamin D serum levels were analyzed by using commercial ELISA kits (Pishgaman Sanjesh- Iran) and an Awareness/Stat Fax 2100 analyzer. Serum 25 (OH) vitamin D was classified according to the established cutoff values (ng/mL): serum 25 (OH) vitamin D concentrations < 20ng/ml deficiency, 20-29ng/ml insufficiency, and ≥ 30ng/ml sufficiency levels [39].

### Nano encapsulated formulation and dairy products manufacture

Ingredients used for generating nanocapsules were: oleic acid as liquid lipid, precirol as solid lipid, vitamin D as the bioactive fatty core, deionized water, and poloxamer 188 as surfactant. All ingredients were homogenized with ultrasound and high tensile stress.

The faculty of Food Science and Technology monitored the Fortification of low-fat dairy products in the Salamat dairy factory. Nutritional information for each 100 g yogurt and milk contained: 56 kcal, sugar-free, protein 7 g, fat 3 g, and trans fatty acids 0.04 g.

The dairy products in the intervention and control groups are quite similar in terms of appearance, taste, and smell. Dairy products including 1500IU of Nano encapsulated vitamin D were received by intervention groups only. The transferring and intake of products (placebo or intervention) were done on the day of production or the next day.

### Statistical analysis

All statistical analyses were conducted with SPSS version 16.0 for Windows (Armonk, NY: IBM Corp.). First, the normal distribution of all variables was assessed using the Kolmogorov-Smirnov test. For the comparison of quantitative variables in two groups, an independent t-test was used. Also, for comparison between groups, a one-way ANOVA (Analysis of Variance) test was used. In the case of non-normal variables, a non-parametric equation was applied. All tests were two-tailed, and *p* < 0.05 was considered statistically significant.

## Results

Among all 306 eligible subjects, 17 participants were excluded from the study due to the following reasons: Unwillingness to continue the study (*n* = 7), serum vitamin D concentration > 30ng/ml at baseline (*n* = 3), consumption of multivitamins or supplements throughout the trial (*n* = 4), pregnancy (*n* = 1), developing a heart arrhythmia (*n* = 1), and traveling (*n* = 1). Finally, 141 subjects out of the 289 subjects were women who entered this pilot study. We put the population who received fortified low-fat dairy products (either yogurt or milk) in the intervention group (*n* = 71), and who received simple low-fat dairy products (either yogurt or milk) in the placebo group (*n* = 70) for analyses.

**Fig. 2:**
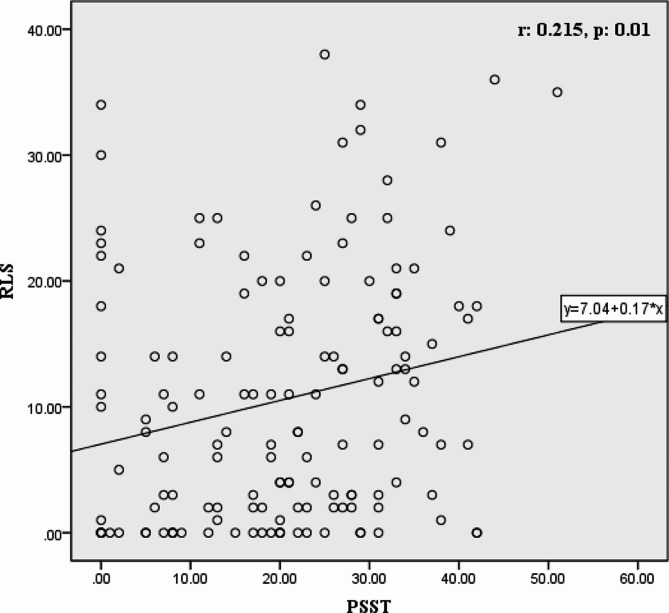
Correlation between RLS and PSST in the intervention group

All 141 women were recruited in this study and classified into three PMS subgroups (mild, moderate, and severe). As shown in Table 1, which was related to pre-intervention, mean serum concentrations of vitamin D in the three PMS subgroups were not significantly different in the intervention group (*p* > 0.05). The mean age and serum level of iron and magnesium did not significantly differ between the three groups of PSST (*p* > 0.05); nevertheless, there was no significant difference in all three PMS subgroups of the control group (Table 1). In this regard, linear regression was performed to determine the correlation between RLS and PSST and showed that RLS was significantly related to PSST (B: 0.174, *p* = 0.01) (Fig. 2).

**Table 1:** Mean of age, serum vitamin D, iron, magnesium and RLS according to PSST categorize in intervention and control groups

PSST score	< 19	19–28	> 28	*p*-value
Intervention group (*n* = 71)				
Intervention group (*n* = 71)	n: 31	n: 18	n: 22	
**Age (year)**	43.26 ± 7.94*	41.52 ± 8.15	38.75 ± 5.37^a^	0.063
**Serum 25 (OH) D (ng/ml)**	14.1 ± 5.95	14.32 ± 4.52	15.61 ± 4.68	0.56
**Iron (mcg/dL)**	76.94 ± 39.65	84.6 ± 38.35	78.59 ± 41.62	0.39
**Magnesium (mEq/L)**	2.147 ± 0.21	2.144 ± 0.17	2.2 ± 0.2	0.51
**RLS score**	7.6 ± 9.13	10.57 ± 9.61	15.48 ± 10.57^a^	0.031
Control group (*n* = 70)				
Control group (*n* = 70)	n: 26	n: 27	n: 17	
**Age (year)**	42.34 ± 7.13	41.33 ± 9.22	39.01 ± 4.44	0.32
**Serum 25 (OH) D (ng/ml)**	17.13 ± 4.8	16.75 ± 4.55	14.34 ± 6.05	0.16
**Iron (mcg/dL)**	71.69 ± 38.62	80.29 ± 36.46	93.26 ± 45.08	0.2
**Magnesium (mEq/L)**	2.14 ± 0.22	2.13 ± 0.17	2.09 ± 0.21	0.66
**RLS score**	9.26 ± 9.5	8.44 ± 9.11	13.57 ± 8.45	0.15

*: mean ± SD. For comparison between groups, a one-way ANOVA test was used

^a^: PSST 0–19 vs. 19–28 and > 28

**Fig. 3:**
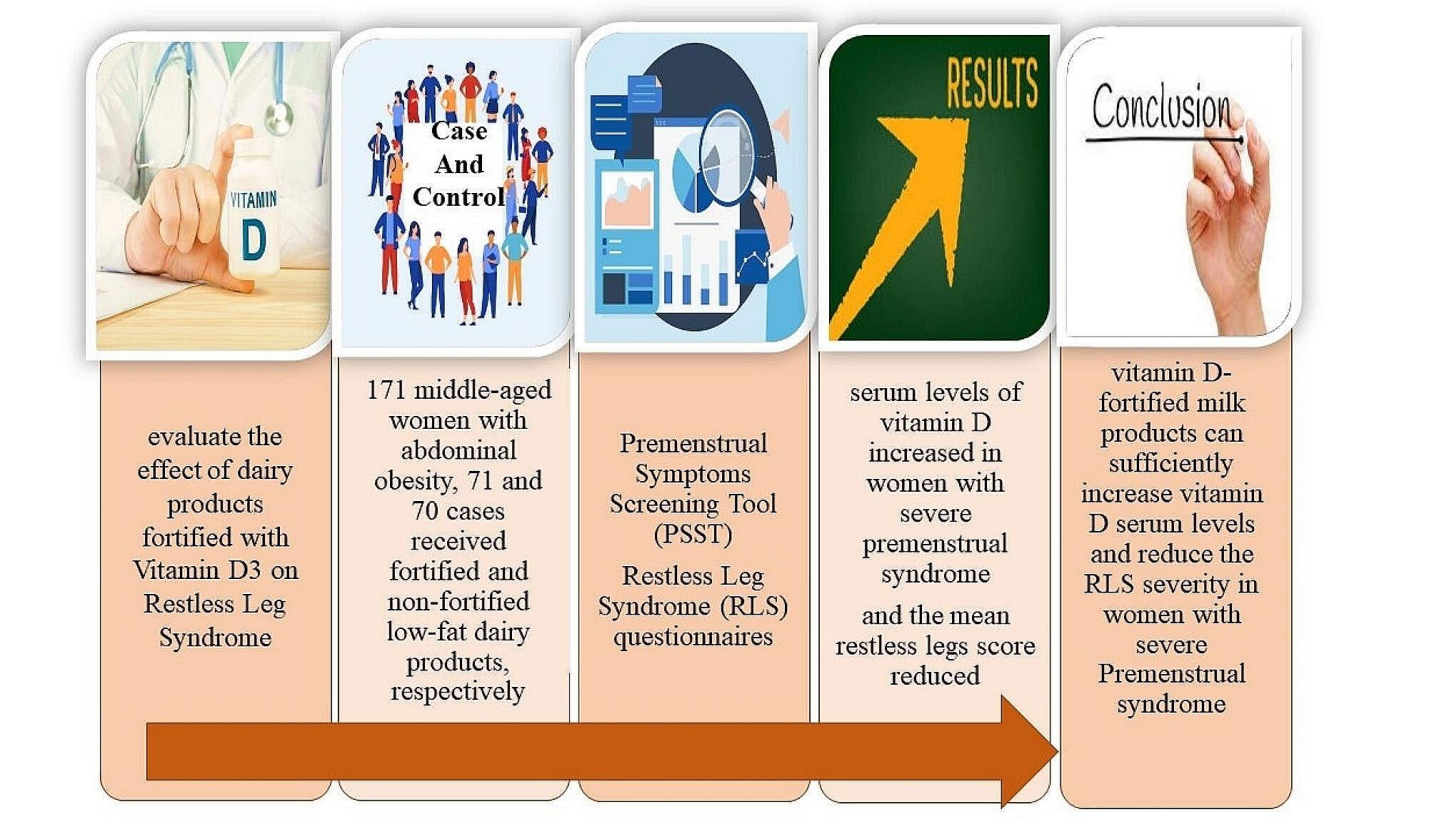
Graphical Abstract

As shown in Table 2, serum levels of vitamin D were evaluated in three subgroups in the intervention group (*p* < 0.05). Moreover, the RLS score was decreased in individuals with PSST > 28 in the intervention group (*p* < 0.05). No significant difference was found in any of the three PMS subgroups in the control group before and after the intervention (Table 3). Regarding Table 4, changes in serum Vitamin D significantly differed between intervention and control groups in all individuals (PSST < 19, PSST 19–28, and PSST > 28) (*p* < 0.05). Both the control and intervention group did not show any significant differences with RLS scores in the three PMS subgroups (*p* > 0.05). We summarized the paper in graphical abstract (Fig. 3).

**Table 2:** Role of fortified dairy product consumption on vitamin D and RLS in intervention group

		Before intervention	After intervention	*p*-value
**Serum 25 (OH) D (ng/ml)**	< 19	14.1 ± 5.95	19.94 ± 6.61	< 0.001
	19–28	14.32 ± 4.52	21.16 ± 5.23	< 0.001
	> 28	15.61 ± 4.68	21.01 ± 4.58	< 0.001
**RLS score**	< 19	7.3 ± 7.93	6.4 ± 7.45	0.33
	19–28	10.82 ± 10.61	8.17 ± 10.52	0.29
	> 28	15.27 ± 12.21	11.36 ± 10.68	0.048

**Table 3:** Role of non-fortified dairy product consumption on vitamin D and RLS in control group

		Before intervention	After intervention	*p*-value
**Serum 25 (OH) D (ng/ml)**	< 19	17.13 ± 4.8	16.1 ± 4.44	0.14
	19–28	16.75 ± 4.55	16.34 ± 4.7	0.2
	> 28	14.34 ± 6.05	14.2 ± 6.68	0.85
**RLS score**	< 19	9.75 ± 9.5	7.65 ± 7.36	0.38
	19–28	8.95 ± 9.11	8.04 ± 8.56	0.77
	> 28	13.57 ± 8.45	11.1 ± 8.18	0.14

**Table 4:** Changes of fortified dairy product consumption on Vitamin D and RLS in intervention and control groups

		Intervention	Control	*p*-value
**Serum 25 (OH) D (ng/ml)**	< 19	5.835 ± 3.1	-1.03 ± 4.2	< 0.001
	19–28	6.83 ± 2.55	-0.4 ± 3.19	< 0.001
	> 28	5.39 ± 2.8172	-0.14 ± 3.51	< 0.001
**RLS score**	< 19	-0.75 ± 5.48	-1.61 ± 9.36	0.67
	19–28	-2.001 ± 6.21	0.44 ± 7.8	0.26
	> 28	-3.9 ± 12.21	-2.47 ± 7.01	0.62

## Discussion

To the best of our knowledge, this is the first parallel totally-blinded randomized clinical trial evaluating the effectiveness of vitamin D-fortified dairy products in improving RLS in women with PMS. In the current study, we found that fortified low-fat dairy products including 1500 IU Nano-encapsulated vitamin D_3_ resulted in a significant improvement of RLS in participants with severe PMS.

Vitamin D is a neuro-steroid that can cross the blood-brain barrier [40]. It might affect the regulation of the synthesis of acetylcholine, dopamine, serotonin, and gamma-aminobutyric acid [41]. Previous studies have confirmed that Vitamin D receptors are present in neurons and glial cells, which are activated by vitamin D, and finally results in some mechanisms including, differentiation, regulation of Ca^2+^ ions, homeostasis, neurotrophins modulation, and release and activation of major cerebral hormones and enzymes for neurotransmitter metabolism [3, 42].

There is accumulating evidence that vitamin D deficiency causes dopaminergic dysregulation, which results in RLS [43, 44]. In a clinical trial on 35 people with idiopathic RLS, 21 had vitamin D deficiency and received 50,000 units of vitamin D weekly for two months (study group). They suggested that the severity of vitamin D deficiency is related to the disease severity [41]. Another study on 12 patients with primary RLS and vitamin D deficiency, revealed a significant association between 28,000 units of vitamin D weekly or 200,000 units of vitamin D monthly intramuscular with a maintenance dose of 400 units per day and decreasing in RLS severity [6]. Buratti et al. in a case study on a woman with Turner syndrome, RLS, and vitamin D deficiency showed that vitamin D supplementation decreases RLS severity [45]. Wali et al. designed a 12-week randomized, placebo-controlled trial on RLS patients, and there were 17 and 18 participants in the vitamin D (received 50,000 IU caplets orally) and placebo groups, respectively. Their results demonstrated that vitamin D supplementation did not improve RLS symptoms [46], which is inconsistent with our findings. A possible explanation is the dose of vitamin D administration in these two investigations and also participants had normal vitamin D levels before the intervention. The primary sources of vitamin D are skin sunlight exposure and dietary intake including, fortified dairy products, oily fish, some mushrooms, and supplements [25]. The uptake of vitamin D through direct sun exposure has been decreased due to the concern of melanoma and premature skin aging, and reduced time spent outdoors at midday [23]. Therefore, the importance of dietary intake of vitamin D or supplements has been highlighted these days.

RLS is commonly observed in women rather than men. It is hypothesized that high estrogen and progesterone levels are contributed to RLS pathogenesis in pregnancy [47]. It has been previously suggested that the PMS rate is elevated in RLS patients [48]. Also, we included subjects with a high BMI in the study because obesity and high BMI are directly related to an increased risk of RLS [10, 49, 50]. The availability of dopamine D2 receptor in brain decreases in obese people [11].

High dietary vitamin D consumption may decrease PMS through several possible mechanisms including: changing in calcium concentrations, the oscillations of cyclic sex steroids, and neurotransmitter action [18, 51]. Earlier studies have shown that the administration of calcium and dairy products alone or with vitamin D significantly reduced the PMS severity [52, 53]. Sleep disturbance such as RLS is related to PMS [54]. So, this study showed that high vitamin D consumption may decrease RLS by decreasing PMS severity. Furthermore, vitamin D intake can protect dopaminergic pathways and enhance the ability of developing neurons to release dopamine [27, 55]. Thus, it reduces negative effects of obesity on RLS.

In the present study, we used nanotechnology as a novel method in the fortification industry, especially for fat-soluble components, which made this study more qualified. This report is the first that the effect of low-fat yogurt and milk fortified by 1500 IU Nano encapsulated vitamin D_3_ RLS in women with PMS is evaluated.

Some limitations need to be considered in explaining our findings: the small sample size and short follow-up duration. Also, this was a questionnaire-based study there were fewer options for participants to provide answers, which reflect their true feelings. Other large randomized controlled trials are required to validate the current findings and assess the longer-term influence of vitamin D as a fortified ingredient in dairy products.

## Conclusion

We have found that intake of low-fat dairy products fortified with 1500 IU nano encapsulated vitamin D_3_ can improve RLS in women with severe RLS who did not have sufficient levels of vitamin D. Our findings present a promising prospect for future investigations and the development of effective treatment options for individuals with similar conditions. However, further studies are needed to confirm these findings.

## Data Availability

The datasets used and/or analyzed during the current study available from the corresponding author on reasonable request.

## Abbreviations

PSST: Premenstrual Symptoms Screening Tool
RLS: Restless Legs Syndrome
PMS: Premenstrual Syndrome
BMI: Body Mass Index
SUVNIA: Survey of ultraviolet intake by nutritional approach
IDF: International Diabetes Federation
WC: Waist Circumference
